# Efficacy and safety of Orelabrutinib-based regimens in diffuse large B-cell lymphoma: a single-center retrospective analysis

**DOI:** 10.1007/s10238-023-01231-w

**Published:** 2023-11-04

**Authors:** Ruowen Wei, Yingying Wu, Shan Jiang, Ao Zhang, Lu Zhang, Ling Liu, Yadan Wang, Min Zhang, Heng Mei, Fang Liu, Linghui Xia, Guohui Cui, Jun Fang

**Affiliations:** Institute of Hematology, Union Hospital, Tongji Medical College, Huazhong University of Science and Technology, 1277 Jiefang Avenue, Wuhan, 430022 China

**Keywords:** Diffuse large B-cell lymphoma, Orelabrutinib

## Abstract

Currently, combining chemotherapy with Bruton tyrosine kinase inhibitors (BTKi) has demonstrated significant effectiveness in treating patients with diffuse large B-cell lymphoma. Orelabrutinib is a second-generation BTK inhibitor, and presently, there have been few reports of Orelabrutinib being used to treat DLBCL. We conducted a retrospective investigation to explore the safety and efficacy of Orelabrutinib in combination with chemotherapy or immunotherapy. The study comprised 19 patients with a median age of 61 years. The overall response rate (ORR) was 89.5% with a complete response (CR) rate of 73.7% and a partial response rate (PR) of 15.8%. The estimated 2-year overall survival (OS) and progression-free survival (PFS) rates were 78.6% (95%CI, 59.8%–100%) and 72.2% (95% CI, 52.4%–99.6%), respectively, with a median follow-up time of 11 months (range 2–24). The most prevalent grade 3 or 4 adverse events (AEs), neutropenia (52.6%), anemia (36.8%), thrombocytopenia (26.3%), febrile neutropenia (26.3%), and lung infection (10.5%), were the most common. Our results reveal that Orelabrutinib is an effective therapy for DLBCL patients. Furthermore, our first investigation of the Orelabrutinib application lays a foundation for larger retrospective studies.

**Supplementary Information:**

The online version contains supplementary material available at 10.1007/s10238-023-01231-w.

## Introduction

Diffuse large B-cell lymphoma (DLBCL) is the aggressive and most prevalent subtype of non-Hodgkin lymphoma (NHL). It accounts for 30–58% of non-Hodgkin lymphoma, with a median age of 60 and approximately 30% of patients over the age of 75 [1–3]. The first-line therapy for DLBCL is the R-CHOP regimen which comprises rituximab, cyclophosphamide, doxorubicin, vincristine, and prednisone. R-CHOP regimen can cure more than half of patients with DLBCL [3–5]. However, around one-third of patients with R-CHOP failure have refractory/relapse (R/R) disorders and dismal prognoses [6]. Moreover, certain subtypes of DLBCL, such as double-expressor lymphoma (DEL) and non-germinal center B-cell–like (non-GCB) DLBCL, lead to worse outcomes [3, 7]. The 5-year overall survival (OS) and the 5-year progression-free survival (PFS) rates for patients with DEL treated with the RCHOP regimen were 30%–36% and 27–32%, respectively [8, 9]. The activated B-cell-like (ABC) subtype showed poorer outcomes, with an estimated 3-year PFS of about 40% compared to 75% for the GCB subtype [10, 11]. Furthermore, the prognosis is worse in elderly patients due to factors such as intolerance to chemotherapy, comorbidities, and increasing incidences of ABC DLBCL and high-grade B-cell lymphoma (HGBL) [12–16].

Many attempts have been made based on R-CHOP to outperform the traditional regimen. Increasing the intensity of chemotherapy is one option. Compared to R-CHOP, R-ACVBP (rituximab, doxorubicin, cyclophosphamide, vindesine, bleomycin, and prednisone) improved OS and PFS in patients with an age-adjusted International Prognostic Index (IPI) score of 1. However, the increased toxicity of chemotherapy hinders its use in clinical applications [17]. DA-EPOCH-R (dose-adjusted etoposide, prednisone, vincristine, cyclophosphamide, and doxorubicin with rituximab) has shown promising results in patients with MYC-rearranged aggressive B-cell lymphomas [18]. However, a comparison of DA-EPOCH-R and R-CHOP revealed that the former was associated with greater toxicities and thus did not improve PFS or OS in the whole cohort [19].

Another approach that could be explored is the combination of novel medication with chemotherapy. Several new drugs have been evaluated in recent years, and BTK inhibitors (BTKi) are proving to be quite beneficial. Ibrutinib, a first-generation BTKi, has shown promise in recent clinical studies when combined with different regimens in improving the prognosis of DLBCL patients [5, 6, 20–22]. Ibrutinib plus R-CHOP improved event-free, progression-free (EFS), PFS, and OS in newly diagnosed patients with DEL younger than 60 years compared to placebo plus R-CHOP. However, ibrutinib combined with R-CHOP was related to deteriorating EFS, PFS, or OS in patients older than 60 years. Patients with advanced age may benefit less from BTKi due to ibrutinib-related side effects and limited exposure to immunochemotherapy [21].

Orelabrutinib, a new generation of BTKi, provides more selective and lasting inhibition of BTK with fewer off-target effects. This results in fewer adverse reactions and improved safety [23]. Ibrutinib has demonstrated long-term efficacy as a single agent in the treatment of R/R mental cell lymphoma (MCL), with a median follow-up period of 15.3 months and an overall response rate (ORR) of 68% [24]. Orelabrutinib monotherapy was reported to achieve 87.6% of ORR in R/R MCL in a median follow-up period of 15.0 months [25]. In the case of R/R chronic lymphocytic leukemia/small lymphocytic lymphoma (CLL/SLL), ibrutinib monotherapy achieved an ORR of 71% after a median follow-up time of 20.9 months [26]. On the other hand, Orelabrutinib monotherapy achieved an ORR of 92.5% after a 32.3-month median follow-up time [27]. While Orelabrutinib has gained prominence as a therapy for DLBCL in recent conferences [28, 29], its safety and efficacy remain unclear due to the lack of available information. Thus, the purpose of this study was to investigate the efficacy and safety of Orelabrutinib-based regimens in the treatment of DLBCL.

## Materials and methods

### Study population and ethical clearance

This was a single-center, retrospective study of 19 individuals diagnosed with DLBCL between August 2020 and August 2022 using the 2016 WHO criteria [30]. This research included 17 newly diagnosed patients and two patients who had a recurrence of initial malignancy. Biopsies of lymph nodes or other mass tissues, bone marrow biopsy and morphologic assessments, next-generation sequencing (NGS), immunohistochemistry, and positron emission tomography/computed tomography (PET/CT) scans were the most commonly used diagnostic tools. Prior to sample collection and treatment, all patients provided informed written consent. The Ethics Committee of Union Hospital, Tongji Medical College, and Huazhong University of Science and Technology approved the protocols of the study (Ethics Approval No. UHCT230616). All study procedures were carried out following the Helsinki Declaration. Hans classification confirmed the GCB and non-GCB subtypes using immunohistochemistry. Some patients were genotyped using the seven “LymphGen genotyping" model [31], while those who did not meet the classification requirements were categorized as "others." Patients with HGBL were excluded using fluorescent in situ hybridization (FISH).

### Treatment regimens

Treatment in combination with Orelabrutinib included chemotherapy-based (chemo-based) and chemotherapy-free (chemo-free) therapy. Orelabrutinib was administrated orally at a dose of 150 mg/day from day 1 to day 21 of the chemotherapeutic cycles. Chemo-based treatments were as follows: R-CHOP, R-miniCHOP, R-CDOP, R-miniCDOP, and R-GDP (gemcitabine, dexamethasone, and cisplatin). These regimens were applied in twelve patients. Rituximab plus MTX or TMZ or MA (MTX and cytosine arabinoside) were applied or added in four of five patients who had central nervous system (CNS) involvement or PCNSL. Patients above the age of 75 were given chemo-free therapy (OR2 regimen). They were given rituximab (375 mg/m^2^) intravenously on day + 1, 25 mg of lenalidomide orally on days 2–15, and 150 mg of Orelabrutinib orally and consistently throughout each 21-day cycle. A detailed treatment plan for each patient is provided in a supplemental table. Specific usages were referred to National Comprehensive Cancer Network (NCCN) or Chinese Society of Clinical Oncology (CSCO) guidelines [32, 33]. The initial dosages of therapy were determined above and adjusted based on the tolerance and therapeutic response of the patients.

### Supportive care

Compound sulfamethoxazole (TMP 0.16 g/SMX 0.8 g, daily) was administered to all patients to prevent pneumocystis pneumonia. Valacyclovir (0.5 g, twice daily) was administered for the prevention of herpes zoster. Entecavir (0.5 mg, daily) was used to prevent hepatitis B virus reactivation. For patients with bone marrow involvement, poor performance status, or older than 65 years, pegylated recombinant human granulocyte colony-stimulating factor (PEG⁃rhG⁃GSF, 6 mg once a week) was used to prevent neutropenia. A secondary prevention strategy was adopted for the remaining patients, PEG⁃rhG⁃GSF would be applied in patients who had not received prophylactic PEG⁃rhG⁃GSF, but had a history of neutropenia in the previous treatment cycle [34]. If necessary, erythropoietin (EPO) or thrombopoietin (TPO) treatments and red blood cell (RBC) or platelet (PLT) transfusions were given by the physicians according to the guidelines.

### Therapeutic evaluation

PET/CT was used by all patients for the evaluation of their interim or final treatment response. According to the 2014 Lugano Classification [35], treatment responses were classified as complete response (CR), partial response (PR), stable disease (SD), or progressive disease (PD). The ORR refers to the percentage of patients who achieve CR and PR. The time between the first response to treatment and disease progression or death was defined as the responder’s duration of response (DOR). The interval from the date of diagnosis and the date of death from any cause or the final day of follow-up was defined as the overall survival (OS). The time between diagnosis and relapse, death from any cause, or the last follow-up day was defined as progression-free survival (PFS). The Common Terminology Criteria for Adverse Events version 5.0 was used to evaluate the adverse events (AEs) of treatment. The ORR was the primary endpoint of the study, while CR rate, DOR, OS, and PFS were the secondary endpoints.

### Statistical analysis

The patient’s demographic and clinical data were analyzed using descriptive statistical analysis. Continuous variables were expressed as medians and ranges. Classification variables were examined using Fisher’s exact test for subgroup analysis. The log-rank test was used to estimate OS and PFS using Kaplan–Meier analysis. The statistical work was carried out in SPSS version 25.0 (SPSS Inc., Chicago, IL, USA) and R Studio version 4.3.0 (R Development Team).

## Results

### Patient characteristics

We included 19 DLBCL patients with a median age of 61 years (range 36–83). The median duration of follow-up time was 11 months (range 2–24). The basic information is summarized in Table 1. A total of 17 patients were newly diagnosed with DLBCL and two suffered R/R disease (Patient 16 and Patient 18). Two patients were diagnosed with PCNSL (P16 and P19), and three patients had CNS involvement (P11, P15, and P18). Two patients were diagnosed with EBV-positive DLBCL (P9 and P12). Two patients had CD5-positive DLBCL (P15 and P2) in which one patient (P2) was accompanied with follicular lymphoma (FL). The Richter's transformation existed in one patient (P19), and one patient was transformed from FL (P6). The number of patients with GCB and non-GCB lymphomas was 6 (31.6%) and 13 (68.4%), respectively. Ten patients (10/19, 52.6%) were identified as DEL. At the time of diagnosis, samples from 11 individuals were subjected to NGS. The probabilities of having A53, MCD, and other genotypes were 27.3% (3/11), 18.2% (2/11), and 54.5% (6/11), respectively. Chemotherapy combined with Orelabrutinib was administered to fifteen patients. The OR2 regimens were administered to four individuals aged 75 and over. Detailed information is provided in the supplemental table.

**Table 1:** Basic information of DLBCL patients

Characteristics	Whole cohort
Number of patients	19
Median age, year (range)	61 (36–83)
*Gender*	
Male	10 (52.6)
Female	9 (47.4)
*ECOG score*	
0–1	11 (57.9)
≥ 2	8 (42.1)
*B symptoms*	
No	9 (47.4)
Yes	10 (52.6)
*LDH at diagnose*	
Normal	11 (57.9)
Elevated	8 (42.1)
*Lugano stage*	
I–II	7 (36.8)
III–IV	12 (63.2)
*IPI*	
0–2	8 (42.1)
3–5	11 (57.9)
*Cell of origin*	
GCB	6 (31.6)
Non-GCB	13 (68.4)
*Double-expressor lymphoma*	
No	9 (47.4)
Yes	10 (52.6)
*Bone marrow involved*	
No	15(78.9)
Yes	4 (21.1)
*CNS involved*	
No	14 (73.7)
Yes	3 (15.8)
PCNSL	2 (10.5)
*Extranodal involvement*	
No	4 (21.1)
Yes	15 (78.9)
*Number of extranodal sites*	
< 2	10 (52.6)
≥ 2	9 (47.4)
Median duration of response, months (range)	7 (1–21)
Median follow-up time, months (range)	11 (2–24)

Data are presented as *n* (%) unless indicated otherwise

*ECOG* Eastern Cooperative Oncology Group; *LDH* lactate dehydrogenase; *IPI* the International Prognostic Index; *CNS* central nervous system; *GCB* germinal center B-like; *non-GCB* non-germinal center B-like; and *R/R* relapse or refractory

### Treatment prior to Orelabrutinib-based regimen

Eight patients received initial chemotherapy prior to Orelabrutinib-based therapy. Treatment regimens before Orelabrutinib-based therapy were as follows: R-CHOP, DA-EPOCH-R, R-CDOP (rituximab, cyclophosphamide, liposomal doxorubicin, vincristine, and prednisone), R-CHOEP (R-CHOP plus etoposide), and R-CDOEP (R-CDOP plus etoposide). The majority of patients were treated with R-CHOP. Patients with DEL or IPI score of 3–5 would opt for R-CHOEP or DA-EPOCH. R-CDOP or R-CDOEP was administered to patients who had cardiac insufficiency. Patients with primary central nervous system lymphoma (PCNSL) were administered with methotrexate (MTX, 3.5 g/m2/day on day 1) and temozolomide (TMZ, from day 2 to day 5). Due to inability to tolerate high-intensity chemotherapy, or a desire for better response to chemotherapy, these patients shift to chemotherapy regimens including Orelabrutinib.

### Treatment response

**Fig. 1:**
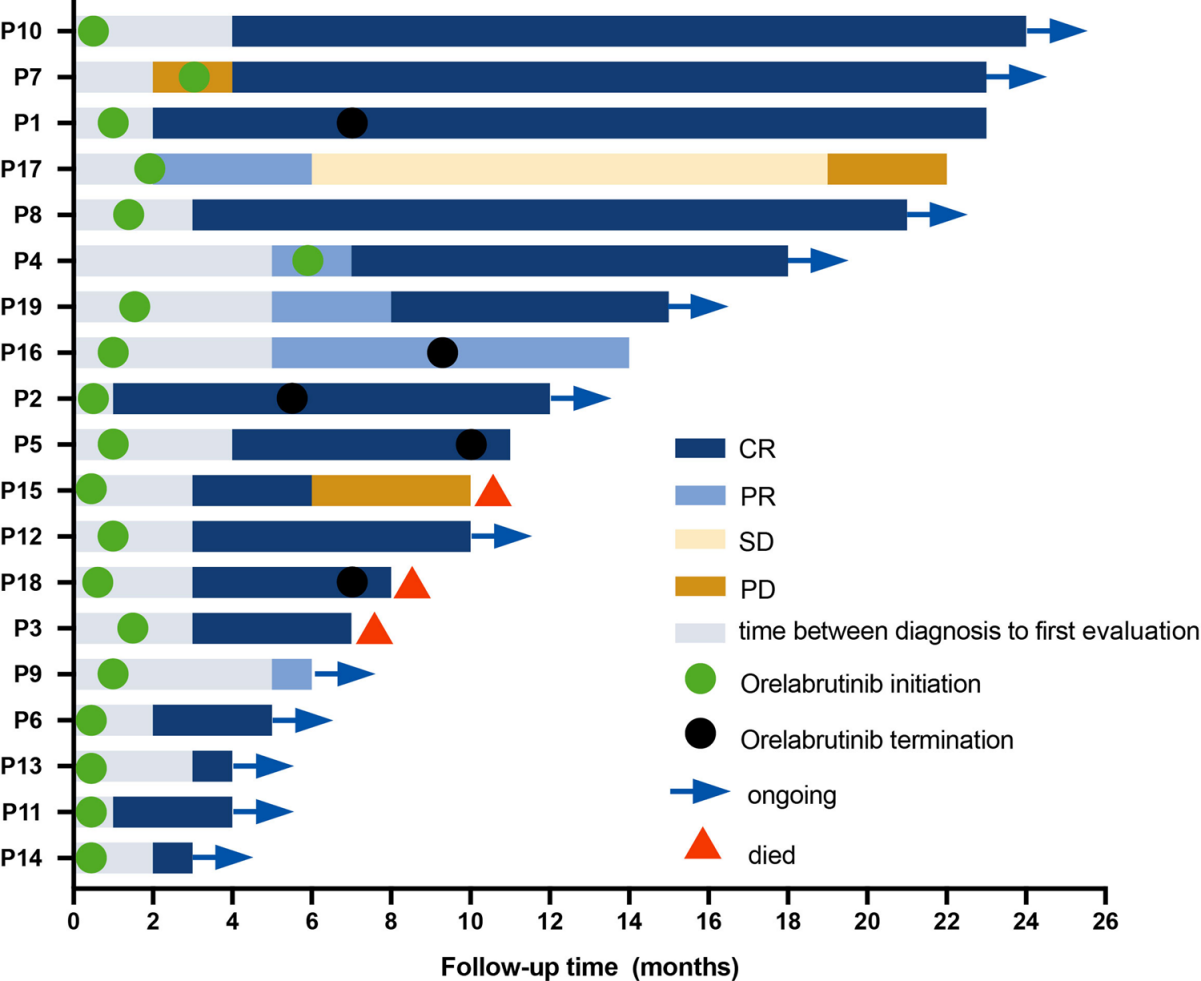
Detailed treatment duration data of patients. Each bar represents a single patient

**Fig. 2:**
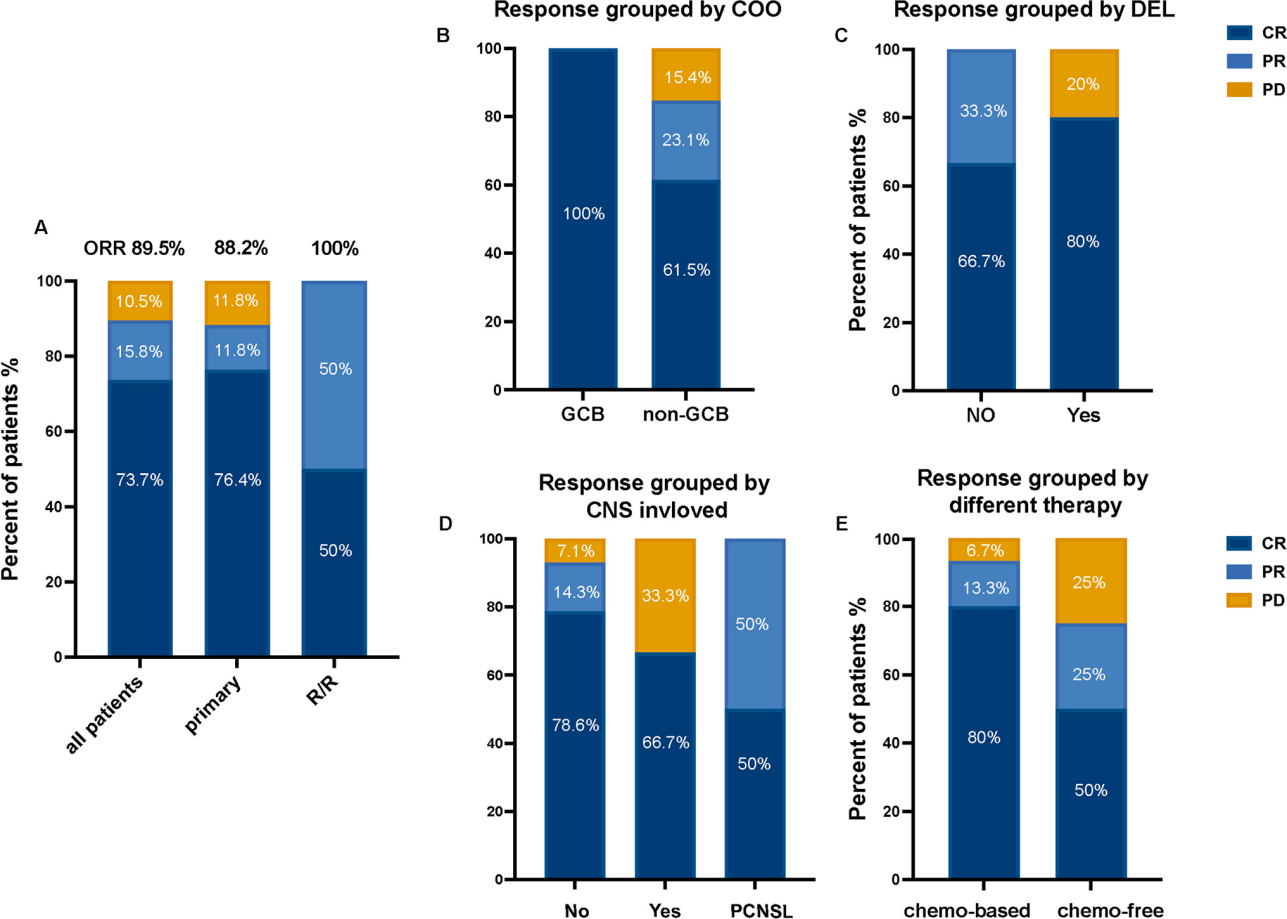
The treatment response in DLBCL patients received Orelabrutinib-based therapy. **A** The ORR was 89.5% in the whole cohort included 17 cases of primary DLBCL and two cases of relapse/refractory (R/R) DLBCL. The ORR was 88.2% in primary DLBCL and 100% in R/R patients, respectively. **B** The treatment response grouped by the cell-of-origin (COO) classification. The ORR was 100% in patients with GCB DLBCL (*n* = 6) and 84.6% in patients with non-GCB DLBCL (*n* = 13), respectively. **C** The treatment response grouped by double-express lymphoma (DEL). The ORR was 100% and 80.0% in patients without (*n *= 9) or with (*n* = 10) DEL, respectively. **D** The treatment response grouped by patients without (*n* = 14) and with (*n* = 5) CNS involvement, the ORR was 92.8% and 80.0%, respectively. **E** The treatment response grouped by different types of therapy. The ORR was 93.3% in patients who were treated with chemotherapy-based regimen plus Orelabrutinib (*n* = 15) and was 75% in patients received chemotherapy-free regimens containing Orelabrutinib (*n* = 4). *Abbreviations*: ORR, overall response rate; CR, complete response; PR, partial response; PD, progressive disease; and chemo, chemotherapy

Detailed treatment duration of patients is presented in Fig. 1. The ORR for all patients was 89.5%, 88.2% for newly diagnosed patients, and 100% for R/R patients (Fig. 2A). The median DOR in this group of evaluable cases was 7 months (range 1–21). Subgroup analyses based on Hans’ classification, DEL, CNS involved, and different methods of treatment. The ORR was 100% in GCB DLBCL patients and 84.6% in non-GCB DLBCL patients (*P* = 1.00, Fig. 2B). The ORR for patients without and with DEL was 100% and 80.0%, respectively (*P* = 0.47, Fig. 2C). The ORR in patients without CNS involvement (*n* = 14), with CNS involvement (*n* = 3), and PCNSL (*n* = 2) was 92.9%, 66.67%, and 100%, respectively (*P* = 0.354, Fig. 2D). Patients who received chemo-based regimens plus Orelabrutinib (*n* = 15) had an ORR of 93.3%, whereas patients that received chemo-free regimens with Orelabrutinib (*n* = 4) had an ORR of 75% (Fig. 2E). In patients with EBV-positive DLBCL, one achieved CR and one achieved PR. One patient with CD5-positive DLBCL achieved CR and one patient had PD. The patients with Richter's transformation or transformed from FL achieved CR. Patients of A53, MCD, and other genotypes had ORRs of 100% (CR, 33.33%), 50% (CR, 50%), and 100% (CR, 83.33%), respectively (*P* = 0.188).

### Reduction of Orelabrutinib

One patient was suspended from taking Orelabrutinib until the last follow-up time due to bone marrow suppression during chemotherapy. Three patients had their Orelabrutinib dosage reduced, two as a result of taking it with azole antifungal, and another one due to gastrointestinal side effects. Seven individuals ceased treatment with Orelabrutinib due to auto stem cell transplantation (*n* = 1), severe pulmonary infection (*n* = 1), tuberculosis recrudescence (*n* = 1), induction chemotherapy finished (*n* = 2), and death (*n* = 2) (Supplementary Table 1).

### Safety and supportive treatment

Fourteen patients (73.7%) experienced adverse events (AEs) of grade 3 or higher. There were 11 (11/15, 73.3%) and 3 (3/4, 75%) patients who experienced grade 3 or severe adverse events following treatment with Orelabrutinib plus chemotherapy and OR2 regimens, respectively. Hematological AEs were the most frequently reported AEs throughout the sample (Table 2). Neutropenia (52.6%), anemia (36.8%), thrombocytopenia (26.3%), febrile neutropenia (26.3%), and pulmonary infection (10.5%) were the most common grade 3 or 4 adverse events. A 75-year-old man with a history of atrial fibrillation (AF) and radiofrequency ablation developed grade 3 atrial flutter and dysrhythmia. The OR-miniCHOP regimen caused grade 4 pancreatitis in a 76-year-old woman; thus, the therapy was changed to OR2. Their condition improved once they received prompt medical attention. Patients with grade 4 diarrhea improved considerably after 2 days of anti-diarrheal and fluid supplement medication. Three patients experienced grade 5 AEs, including intracranial hemorrhage, encephalitis infection, and DLBCL progression.

**Table 2:** Summary of treatment-related adverse events

Whole cohort (*n* = 19)	Grades 1–2	Grades 3–4	Grade 5
Hematological adverse events			
Anemia	9 (47.4)	7 (36.8)	
Thrombocytopenia	5 (26.3)	5 (26.3)	
Neutropenia	5 (26.3)	10 (52.6)	
Febrile neutropenia		5 (26.3)	
Purpura	2 (10.5)		
Intracranial hemorrhage			1 (5.3)
Non-hematological adverse events			
Pulmonary infection	4 (21.1)	2 (10.5)	
Encephalitis infection			1 (5.3)
Aortic valve disease	4 (21.1)		
Mitral valve disease	4 (21.1)		
Tricuspid valve disease	3 (15.8)		
Atrial flutter		1 (5.3)	
Atrial arrhythmias	3 (15.8)	1 (5.3)	
Pericardial effusion	3 (15.8)		
Pancreatitis		1 (5.3)	
Skin rash	3 (15.8)		
Decreased appetite	3 (15.8)		
Nausea	3 (15.8)		
Vomiting	3 (15.8)		
Diarrhea		1 (5.3)	
Disease progression			1 (5.3)

Data are presented as *n* (%) unless indicated otherwise

G-CSF was used as primary prophylaxis to prevent neutropenia before treatment in five patients, and secondary prevention strategy was used in nine patients. Ten patients were treated with G-CSF due to neutropenia. Three patients were treated with TPO including one patient was treated EPO. Four patients received PLT transfusion, and three received RBC transfusion.

### Relapse and survival

**Fig. 3:**
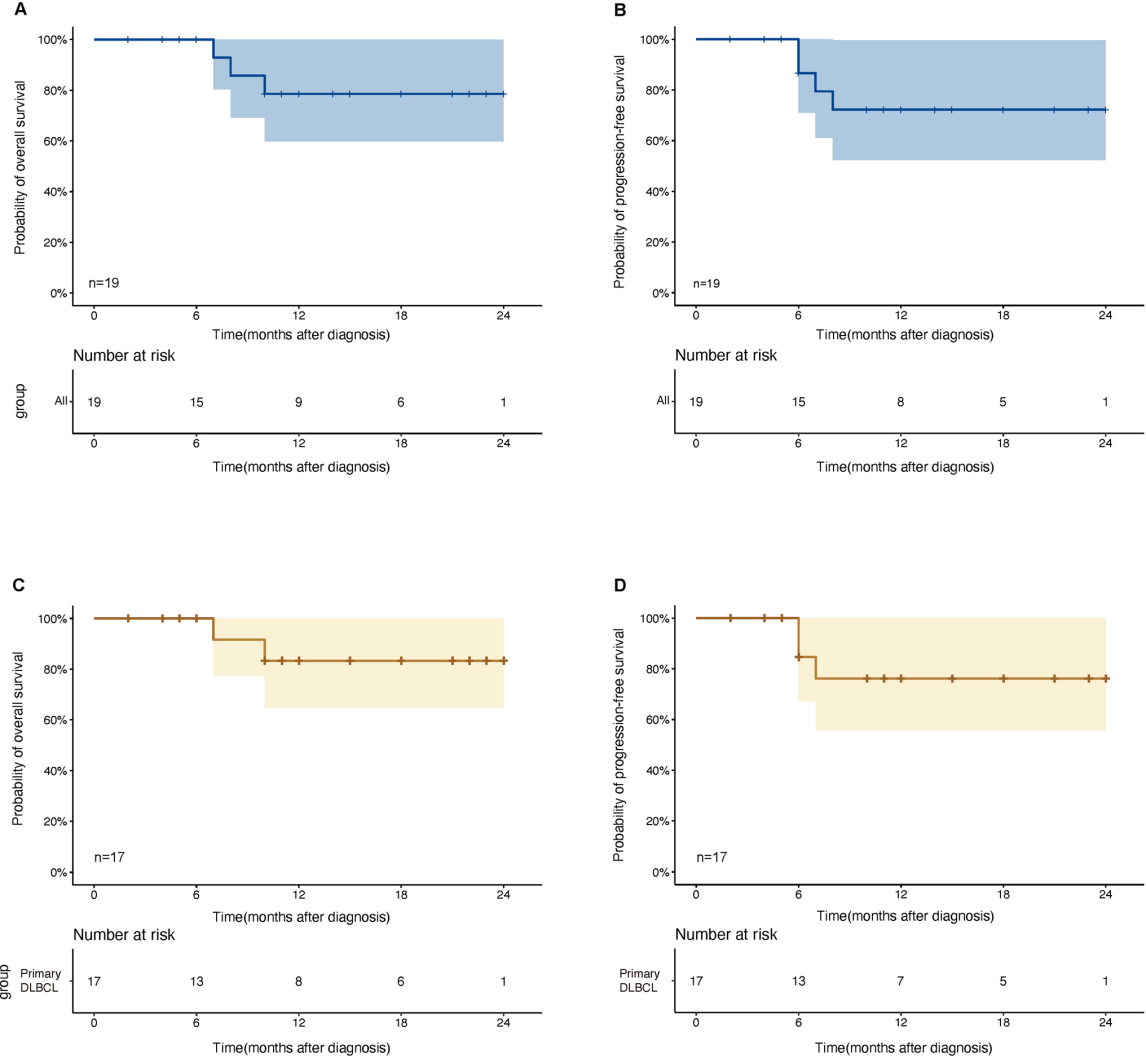
Kaplan−Meier curve for OS and PFS in all patients and primary DLBCL treated with Orelabrutinib-based therapy. **A**, **B** Kaplan−Meier curve for overall survival (OS) and progression-free survival (PFS) in the whole cohort (*n* = 19). The estimated 2-year OS and 2-year PFS rates were 78.6% (95%CI, 59.8%–100%) and 72.2% (95%CI, 52.4%–99.6%) (*n* = 19), respectively. **C**, **D** Kaplan−Meier curve for OS and PFS for newly diagnosed patients (*n* = 17). The estimated 2-year OS and 2-year PFS rates were 83.3% (95%CI, 64.7%–100%) and 76.2% (95%CI,55.8%–100%), respectively

In the entire dataset, three patients died, while four experienced disease progression over a median follow-up time of 11 months (range 2–24). Two patients (one of whom was an R/R patient) died from serious complications of treatment, and one was due to cancer progression. In the case of PCNSL, one patient (50%) died and one (50%) survived. One patient (33.3%) died and two survived (66.7%) with secondary CNS involvement. One patient (7.14%) died among the patients without CNS involvement, with a 92.86% survival rate. The estimated 2-year OS and PFS rates in the whole sample were 78.6% (95% CI, 59.8%–100%) and 72.2% (95% CI, 52.4%–99.6%), respectively (Fig. 3A and B). The 2-year OS and PFS rates for newly diagnosed patients were 83.3% (95% CI, 64.7%–100%) and 76.2% (95% CI, 55.8%–100%) (Fig. 3C and D).

**Fig. 4:**
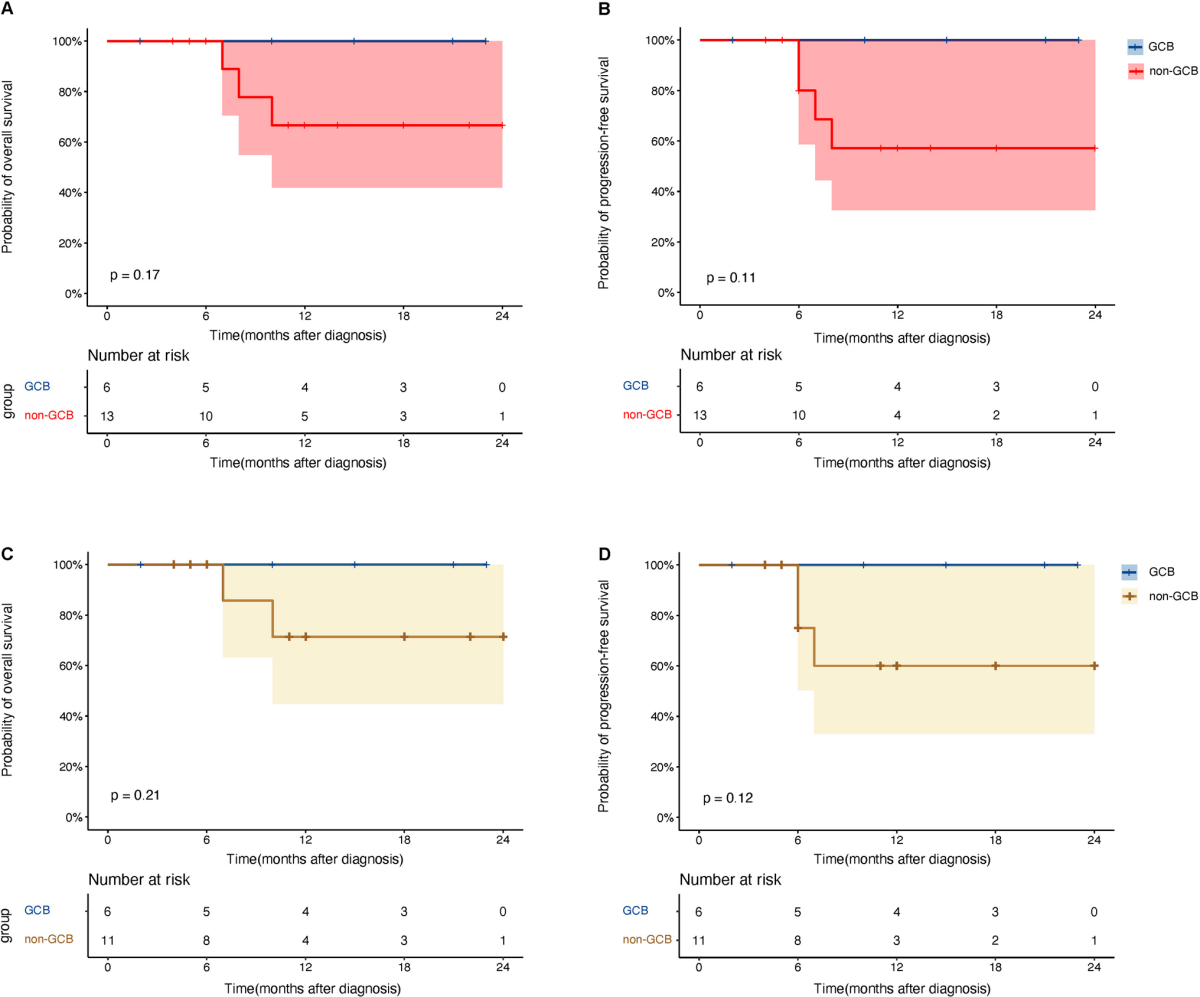
Kaplan−Meier curve for OS and PFS grouped by the cell-of-origin classification in all patients and primary DLBCL treated with Orelabrutinib-based therapy. **A**, **B** Kaplan−Meier curve for OS and PFS in subgroups of all patients. The estimated 2-year OS rates were 100% in the GCB group and 66.7% (95%CI, 42.0%–100%) in the non-GCB group (*P* = 0.17), respectively. The estimated 2-year PFS rates were 100% and 57.1% (95%CI, 32.6%–100%) in the GCB group and non-GCB group (*P* = 0.11), respectively. **C**, **D** Kaplan−Meier curve for OS and PFS in subgroups of newly diagnosed patients. The estimated 2-year OS rates were 100% in the GCB group and 71.4% (95%CI, 44.7%–100%) in the non-GCB group (*P* = 0.21), respectively. The estimated 2-year PFS rates were 100% in the GCB group and 60% (95%CI, 33.1%–100%) in the non-GCB group (*P* = 0.12), respectively

**Fig. 5:**
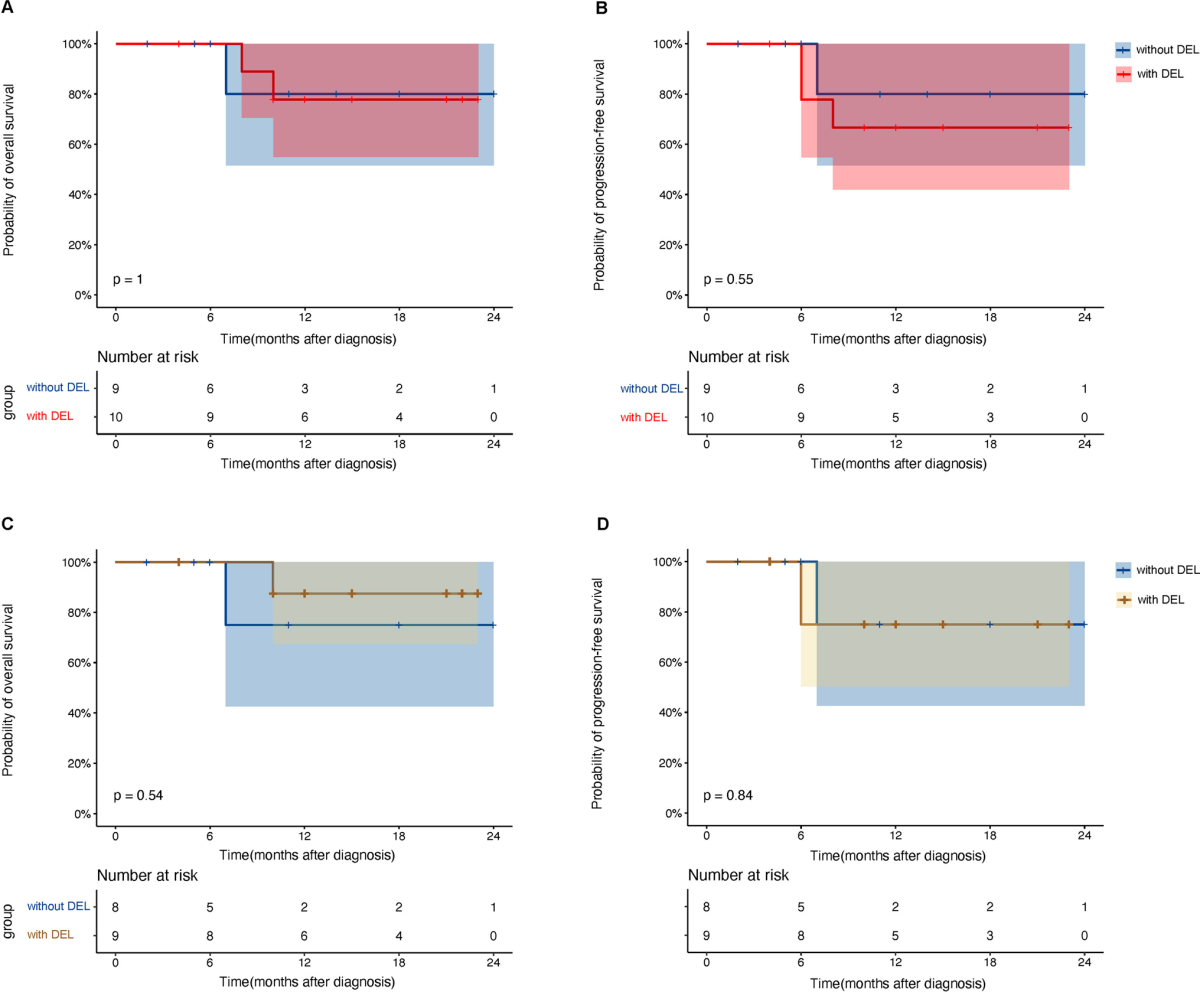
Kaplan−Meier curve for OS and PFS of double-express lymphoma in all patients and primary DLBCL treated with Orelabrutinib-based therapy. **A**, **B** Kaplan−Meier curve for OS and PFS in subgroups of all patients. The estimated 2-year OS rates were 80.0% (95%CI, 51.6%–100%) in patients without DEL and 77.8% (95%CI, 54.9%–100%) in patients with DEL (*P* = 1.00), respectively. The estimated 2-year PFS rates were 80.0% (95%CI, 51.6%–100%) and 66.7% (95%CI, 42.0%–100%) in patients without or with DEL (*P* = 0.55), respectively. **C**, **D** Kaplan−Meier curve for OS and PFS in subgroups of newly diagnosed patients. The estimated 2-year OS rates were 75.0% (95%CI, 42.6%–100%) in patients without DEL and 87.5% (95%CI, 67.3%–100%) in patients with DEL (*P* = 0.54), respectively. The estimated 2-year PFS rates were 75.0% (95%CI, 42.6%–100%) and 75.0% (95%CI, 50.3%–100%) in patients without or with DEL (*P* = 0.84), respectively

In the subgroup analysis, the GCB group had a 2-year OS rate of 100%, whereas the non-GCB group had a rate of 66.7% (95% CI, 42.0%–100%) (P = 0.17, Fig. 4A). The 2-year estimated PFS rates in the GCB group and the non-GCB group were 100% and 57.1% (95% CI, 32.6%–100%), respectively (*P* = 0.11) (Fig. 4B). The OS rate for the newly diagnosed patients in the GCB group was 100%, while the non-GCB group had an OS rate of 71.4% (95% CI, 44.7%–100%) (*P* = 0.21, Fig. 4C). Two-year PFS rates for newly diagnosed patients in the GCB group were 100% and 60% (95%CI, 33.1%–100%) in the non-GCB groups (*P* = 0.12, Fig. 4D). There was no significant difference in estimated 2-year OS (80.0% vs. 77.8%, *P* = 1.00) and PFS (80.0% vs. 66.7%, *P* = 0.55) between patients without and with DEL in the whole group (Fig. 5A and B). Similarly, there was no significant difference in OS (75.0% vs. 87.5%, *P* = 0.54) or PFS (75.0% vs. 75.0%, *P* = 0.84) between newly diagnosed patients with and without DEL (Fig. 5C and D).

## Discussion

The BTK pathway is important in the pathogenesis of DLBCL. BTK inhibitors may play a significant role in this process. It is, therefore, crucial to investigate the combination of R-CHOP and BTKi as a treatment option for DLBCL. Orelabrutinib, as a second-generation BTK inhibitor, has demonstrated potential efficacy in the treatment of CLL/SLL, MCL, and relapsed or refractory Waldenström's macroglobulinemia [25, 27, 36]. However, the therapeutic efficacy of Orelabrutinib against DLBCL remains unknown. Currently, Orelabrutinib has been reported to be successful in the treatment of DLBCL in two international conferences. A study of 14 patients with MCD DLBCL found that the CR rate for the first- and second-line therapies was 75.0% and 66.67%, respectively [29]. The ORR was 90.9%, and the CR rate was 77.3% in a group of 22 untreated non-GCB patients with extranodal invasion. Following a median follow-up of 11 months, three double-expressor lymphoma (DEL) patients developed progressive disease, all of whom survived [28]. In our study, the ORR and CR rates were 89.5% and 73.7% for all patients, 88.2% and 76.4% for newly diagnosed patients, and 100% and 50% for R/R patients (Fig. 2A). In the whole group, the estimated 2-year OS and PFS rates were 78.6% and 72.2%, respectively (Fig. 3A and B). The 2-year OS and PFS rates for newly diagnosed patients were projected to be 83.3% and 76.2%, respectively (Fig. 3C and D).

In the non-GCB group, the ORR and CR rates were 84.6% and 61.5%, respectively. All the patients in the GCB group achieved CR (Fig. 2B). Non-GCB patients experienced a poorer 2-year OS (100% vs. 66.7%, *p* = 0.17) and 2-year PFS (100% vs. 57.1%, *p* = 0.11) than GCB patients, although there was no statistical difference (Fig. 4A and B). Non-GCB patients had a lower OS (100% vs. 71.4%) and PFS (100% vs. 60%) in newly diagnosed patients (Fig. 4C and D). In the Phoenix clinical trial, the 3-year OS and PFS in the intent-to-treat population treated with ibrutinib plus R-CHOP were 85.8% and 70.8%, respectively [21]. Compared to the Phoenix study, the performance status of the patients in this study was worse (ECOG = 2, 42.1% vs. 9.1%), with an increase in the proportion of patients with BM involvement (21.1% vs. 11.9%), DEL involvement (57.9% vs. 43.6%) [37], extranodal involvement ≥ 2 (47.4% vs. 31%), and IPI 3–5 (57.9% vs. 43.6%). Due to the limited number and the heterogeneity of patients in this research, the OS and PFS of the non-GCB group were lower than in the Phoenix study. Large-scale studies are needed to determine the efficacy of Orelabrutinib on non-GCB DLBCL. While DEL patients had slightly lower ORR than that non-DEL patients (80% vs. 100%), their CR rate was higher (80% vs. 66.7%, Fig. 2C). Compared to non-DEL patients, DEL patients achieved a comparable 2-year OS and PFS (Fig. 5). The MYC protein promotes cell proliferation, while the antiapoptotic BCL2 protein contributes to accelerating lymphoma progression by interaction with other oncogenes, especially MYC. Co-expression of them plays a key role in DEL pathogenesis, possibly indicating cumulative genomic change in nuclear factor-kappa B (NF-κB) and the B-cell receptor (BCR) signaling pathway [38, 39]. BTKi can block BCR signaling pathway and NF-κB activation, inhibit malignant proliferation, and induce apoptosis of tumor [40]. Hui Yu et al. demonstrated that Orelabrutinib downregulated BTK phospholipase C-g2 and NF-kB signaling pathway to inhibit the proliferation of ABC-DLBCL cell lines (TMD8 and HBL-1) [41], which co-express MYC and BCL2 [42–44]. Furthermore, Orelabrutinib combined with rituximab displayed promising anti-tumor effects in vitro and in vivo [41]. In the phase 3 PHOENIX trial, DEL patients receiving ibrutinib plus R-CHOP had comparable OS (HR: 1.042, 95%CI: [1.708–1.534], *P* = 0.8338) and EFS (HR: 0.987, 95%CI: [0.608–1.601], *P* = 0.9573) to non-DEL patients. Moreover, ibrutinib plus R-CHOP increased EFS and OS in patients younger than 60 years old with DEL compared to R-CHOP, but there was no significant difference in older patients [37]. BTKi have shown potential benefits in the treatment of DEL patients, but further research is needed to discover its specific mechanism toward DEL, and whether they might aid older DEL patients. Orelabrutinib-based regimens have yielded promising results in the treatment of central nervous system lymphoma (CNSL). The ORR in R/R PCNSL was 60%–86.7% and 100% in newly diagnosed PCNSL [45, 46]. In the current study, the ORR was 66.7% in patients with CNS involvement and 100% PCNSL. Although the sample size in the existing literature is small, our results and other studies demonstrate that Orelabrutinib has significant potential for treating central lymphoma and that large-scale studies are necessary to validate its efficacy.

In our study, the ORR for the chemo-based group was 93.3%, with 80% of them achieving CR (Fig. 2E). The ORR for patients aged 75 or older receiving the OR2 regimen was 75% with a 25% CR rate. Yanan Zhu et al. used BTK inhibitors in combination with rituximab and lenalidomide to treat elderly or unfit patients with DLBCL. The elderly patients with a median age of 75 years had an ORR of 87.5%, with 62.5% achieving CR [47]. Peng-Peng Xu et al. used the IR2 regimen (ibrutinib, rituximab, and lenalidomide) to treat unfit and frail de novo DLBCL patients aged 75 years and older. The overall response rate (ORR) was 66.7%, with 56.7% of the patients achieving complete remission (CR) [48]. The CR rate was lower in comparison with prior studies due to the small number of participants.

Adverse events must be considered when BTK inhibitors are used with chemotherapy. Five clinical trials of Orelabrutinib monotherapy in B-cell malignancies (ICP-CL-00102, ICP-CL-00103, ICP-CL-00104, ICP-CL-00105, and ICP-CL-00106) involving 304 patients revealed that the incidence of grade 3 or higher AEs was 38.8%, and the incidence of grade 3 or higher neutropenia and thrombocytopenia was 11.8% and 8.6%, respectively. In a study of Orelabrutinib monotherapy in treating R/R CLL/SLL, neutropenia, thrombocytopenia, upper respiratory tract infection, and positive urine red blood cells were the most frequent AEs (> 30% of any grade). There were no reports of atrial fibrillation nor secondary malignancies, no patients developed high blood pressure, and only one patient developed diarrhea of 3 grade [49]. Ibrutinib-based treatment caused grade 3 or worse AEs in 70.7% of DLBCL patients, while grade 5 AEs occurred in 4.1% of patients [6]. In the Phoenix study, ibrutinib with R-CHOP resulted in similar treatment-emergent AEs (89.9% vs. 87.1%) similar to placebo plus R-CHOP. However, there were more serious AEs recorded with ibrutinib plus R-CHOP (53.1% vs. 34%) [21]. According to a study of Orelabrutinib plus R-CHOP in non-GCB patients (*n* = 22) with extranodal disease, serious adverse events included febrile neutropenia in three patients and atrial flutter in one patient [28]. In a study of Orelabrutinib plus R-CHOP in MCD DLBCL (*n* = 14), the most common adverse events were decreased counts of platelets, white blood cells, and lymphocytes, and non-hematologic adverse events included urinary and respiratory infections (specific data were not shown) [29]. A study of Orelabrutinib-based regimens for CNSL (*n* = 23) reported 21 AEs, of which six patients experienced grade 3 or more AEs, including leukopenia, thrombocytopenia, and hemoglobin decreases [45]. In our study, 73.3% of the patients under the age of 75 suffered from AEs of grade 3 or higher. Two of them suffered grade 5 AEs and died of intracranial hemorrhage or infection. For patients aged 75 or older, three patients (75%) experienced grade 3 or higher AEs with one experiencing grade 4 pancreatitis and grade 5 progressive disease. One patient (25%) with a history of heart disease developed grade 3 AF. One patient (25%) developed grades 3–4 neutropenia, thrombocytopenia, and anemia. Despite several patients receiving dose reductions due to antifungal medications, no patients with OR2 died from hematologic or non-hematologic AEs. In the study of rituximab, lenalidomide, and BTK inhibitor for elderly or unfit patients with DLBCL by Yanan Zhu et al., the top three most prevalent grades 3–4 AEs were neutropenia (25.8%), infection (19.4%), and rash (9.7%) [47]. In the study of Peng-Peng Xu et al., the common grades 3–4 hematologic AEs were neutropenia (23%), thrombocytopenia (10%), and anemia (7%). Elsewhere, pulmonary infection (23%) and atrial fibrillation (22%) were the most common grades 3–4 non-hematologic AEs [48]. The limited number of elderly patients in our study resulted in a higher frequency of AEs. Several studies have shown that patients taking ibrutinib can develop invasive fungal infections [50–53]. This may be attributed to ibrutinib-induced neutrophil granulocyte dysfunction [54]. It is still unknown how Orelabrutinib affects neutrophils. However, BTK inhibitors share similar metabolic pathways and chemical properties. Since chemotherapy treatments have myelosuppressive effects and BTK inhibitors have side effects, the combination of Orelabrutinib and chemotherapy may enhance the incidence and severity of cytopenia. In clinical trials, AF incidence ranged from 6 to 16%, suggesting that ibrutinib may increase the risk of atrial arrhythmias [55]. Most of the valve dysfunction and arrhythmias observed in our research participants were transient, and heart function was restored in the absence of chemotherapy. However, in older patients with many comorbidities, cardiac disorders, and other non-hematologic AEs can be fatal. Although the OR2 regimen is less myelosuppressive and better tolerated by patients, there are still risks associated with the treatment that must be considered.

In this retrospective study, we preliminarily demonstrated the efficacy of Orelabrutinib in the treatment of DLBCL. It is important to note that this study has the limitations of small sample size, non-uniform treatment regimens, and heterogeneity of patients. Despite this, our study confirmed the preliminary effectiveness of Orelabrutinib-based treatment, which may help patients with CNS involvement and DEL. Besides, preliminary treatment of elderly patients with the OR2 regimen showed promising outcomes. Moreover, our study provides some suggestions and initial evidence for future large-scale prospective clinical studies. Further studies are needed to determine the exact efficacy and safety of Orelabrutinib in treating DLBCL.

### Supplementary Information

Below is the link to the electronic supplementary material.

Supplementary file1 (XLSX 15 kb)

## Data Availability

In accordance with institutional ethics restrictions, the data of the patients are not available in a public repository but are available from the corresponding authors.
